# Paravertebral Ganglioneuroma in Pediatric Age: A Case Report

**DOI:** 10.7759/cureus.63363

**Published:** 2024-06-28

**Authors:** Andreia Lasca, Inês Laia, Raquel Pires Santos, António Dias Carneiro, Daniela Moreira

**Affiliations:** 1 Family Medicine, Unidade de Saúde Familiar Viriato, Serviço Nacional de Saúde, Viseu, PRT; 2 General and Family Medicine, Unidade de Saúde Familiar Viriato, Serviço Nacional de Saúde, Viseu, PRT

**Keywords:** family medicine, case report, pediatric, pediatric care, gait disturbances, oncology pediatrics

## Abstract

Ganglioneuromas (GNs) are rare benign tumors common in the pediatric population. Although mostly asymptomatic, some can cause symptoms, particularly neurological ones. Here, we report a case of a two-year-old male child, who presented changes in gait, an alarming sign, during a child health surveillance appointment. On physical examination, the child presented a “duck” gait pattern, axial and appendicular hypotonia, proximal weakness of the pelvic girdle, and a positive Gowers sign. The child was referred to a pediatric neurology appointment where he underwent neuraxial magnetic resonance imaging, identifying a large and expansive formation in the dorsolumbar transition suggestive of neuroblastoma, considering the age group. However, a computed tomography-guided biopsy revealed it to be a paravertebral GN. Tumor resection was performed, leaving some paravertebral tumor residue. After one year of motor rehabilitation, the child had a normal neurological examination. The child is currently five years old and is undergoing annual clinical and imaging surveillance. This case allows us to reflect on the importance of encouraging children and young people to attend recommended surveillance appointments and reminds us that the rarest situations can occur.

## Introduction

Ganglioneuromas (GNs) are rare, well-differentiated benign tumors that arise from sympathetic ganglion cells, with embryonic origin in the neural crest [1].

This tumor frequently occurs in the posterior mediastinum and abdomen [2]. The incidence of this condition is not well documented, but it is estimated to be about 0.1% to 0.5% of central nervous system tumors [3]. GNs are predominantly found in women with an average age of diagnosis at seven years old [4,5].

GNs are mostly non-producers of hormones, such as catecholamines and vasoactive intestinal peptides. In most cases, due to their slow growth and lack of endocrinological activity, patients are asymptomatic and do not present obvious changes upon neurological examination, which often leads to incidental findings [6]. Although GN are considered benign, their size can increase and cause compression of adjacent structures, blood vessels, and nerves, becoming symptomatic [7].

Treatment involves complete surgical resection when possible, allowing a histological diagnosis [5]. Surgery is performed to relieve symptoms as well as due to the theoretical concern of its rare malignant transformation into a neuroblastoma. It is a tumor with a poor response to chemotherapy and radiotherapy, so surgery remains the best therapeutic option. The prognosis is excellent, although local recurrences have been reported in scarce situations [8-10].

The association between paravertebral GN and scoliosis is even rarer and has only been sporadically reported [2].

In this case report, we present a case of a dorsolumbar paravertebral GN in a two-year-old boy with scoliosis, diagnosed after showing warning signs during a child health surveillance appointment.

## Case presentation

The patient was a two-year-old Caucasian male child who attended a routine appointment with his family doctor. He belonged to a family with school-age children (Duvall’s Family Stage IV) with an upper-middle-class socioeconomic status (according to the adapted Graffar scale). With no relevant personal records, the pregnancy was monitored according to the National Pregnancy Surveillance Standards, with uncomplicated vaginal delivery at 39 weeks, with an Apgar score of 9/10/10, adequate somatometric measurements, and normal neonatal screenings. The child has been followed by his family doctor since birth, with all essential age-specific health screenings recommended in the National Program for Child and Youth Health [11]. No changes in growth curves or warning signs were ever identified. No relevant family history was present, with regular medication. Vaccination was up to date, according to the National Vaccination Plan.

In February 2021, at the two-year appointment, the child’s mother expressed concern about bilateral coxalgia, more pronounced on the right side, evolving over a month. According to the parents, he started walking at 16 months but always walked on tiptoes, never ran nor jumped, and walked up or down stairs independently, and now, his walking was regressing. Regarding motor development, he had cephalic control in the first month, brought his hands to midline around three months, sat with support at four months, sat without support at six months, and crawled at eight months. In language development, the child showed no changes.

On physical examination, he had a waddling gait and a "duck” gait pattern, axial and appendicular hypotonia, proximal weakness of the pelvic girdle, causing a pelvic tilt during walking, and a positive Gowers sign. No abnormalities were detected in the upper limbs. Given the warning signs, it was decided to request a long spine X-ray, as well as to initiate physiotherapy sessions to exclude any false alarm signal due to lack of stimulation, with a short-term reassessment.

**Figure 1:**
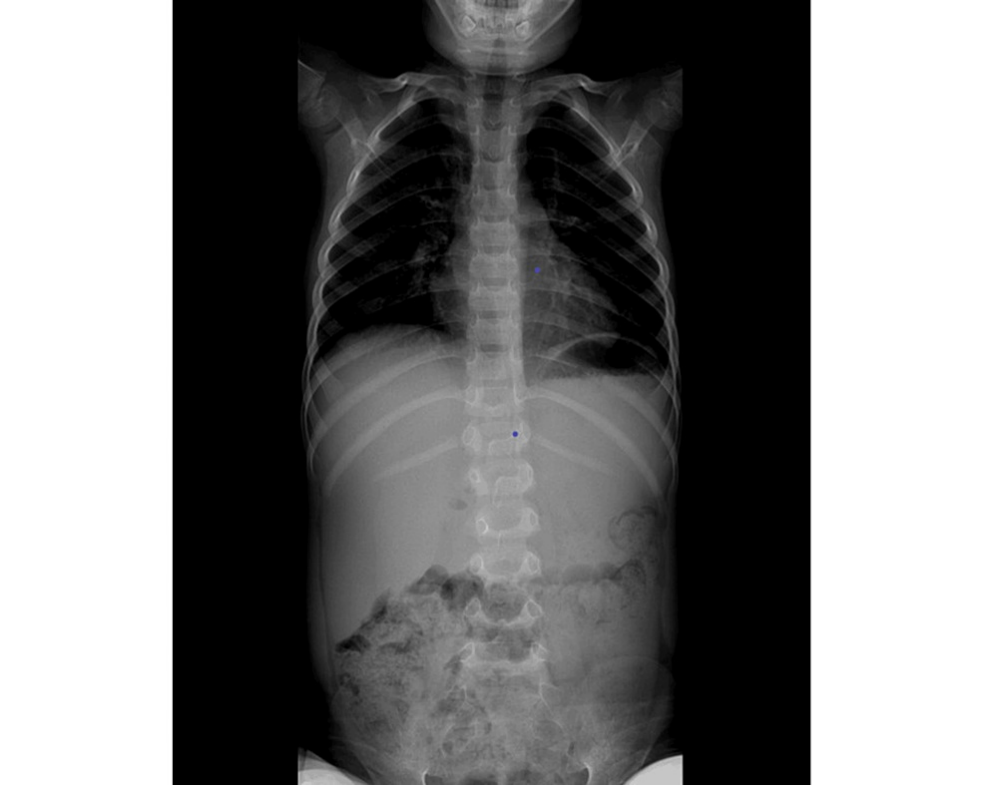
Coronal view of the long spine X-ray. A slight dorsolumbar scoliotic attitude with left convexity centered at L2-L3, although difficult to assess, was not associated with evident body rotation.

After two months of physiotherapy, the mother reported significant regression in walking, with moments when the child did not walk. The long spine X-ray is shown in Figure 1.

**Figure 2:**
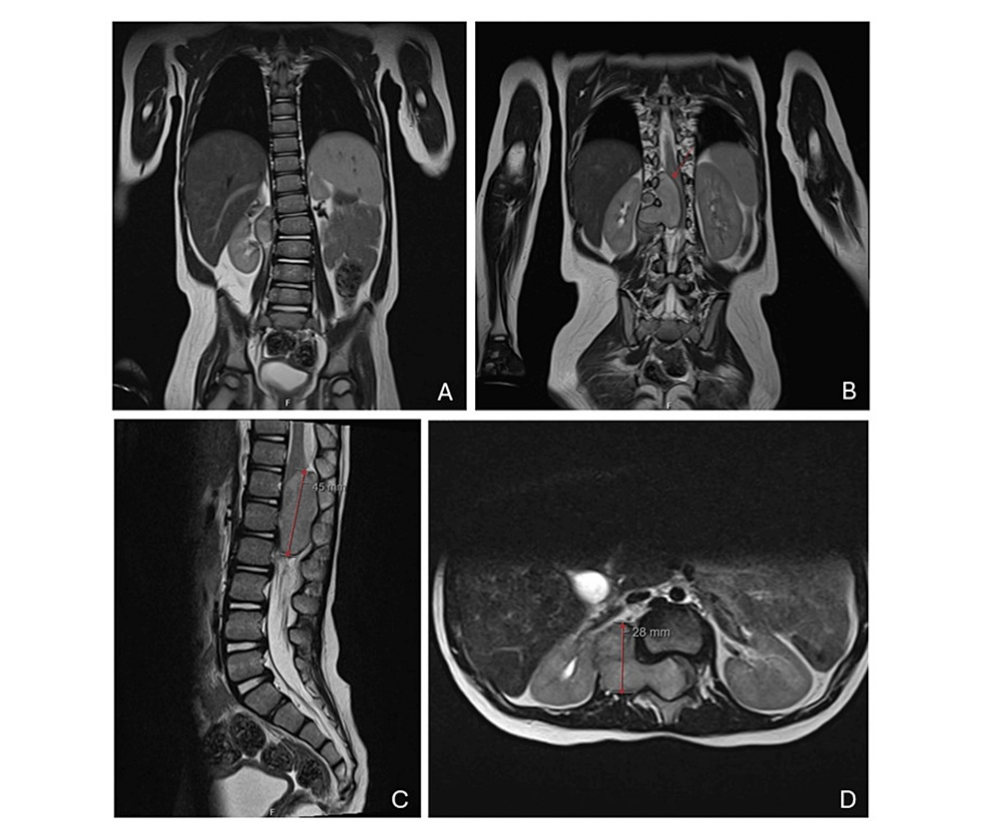
(A) Coronal view showing a slight dorsolumbar scoliosis. (B) Coronal view showing an expansive formation in the dorsolumbar and right lateral paravertebral region. (C) Sagittal view. (D) Axial view of the neuraxial MRI. A large expansive formation in the dorsolumbar and upper lumbar region, occupying both the endocanal and extramedullary compartment and the right lateral paravertebral region, continuing via the intervertebral foramen to the right, especially at the L1-L2 level.

A referral to pediatrics was carried out, and after analyzing the case description, a pediatric neurology appointment was scheduled. In September 2021, the child was evaluated by a pediatric neurologist, and the hypothesis of spinal muscular atrophy was considered. Electromyography of the lower limbs together with extensive lab tests and SMN1 gene sequencing were performed, with normal results. Subsequently, a neuraxis magnetic resonance imaging (MRI) was performed (Figure 2).

The MRI identified a well-defined lesion located at the right lateral paravertebral level, roughly facing the lower endplate of D12 and upper endplate of L3, showing a multi-septated appearance, continuing through the intervertebral foramina on this side, particularly evident at the L1-L2 level expanding the canal and causing a marked deviation to the left and molding of the lower segment of the spinal cord and medullary cone. It did not cause bone destruction. The overall dimensions of the injury were estimated at approximately 45 x 39 x 31 mm (vertical (V) x transversal (T) x anteroposterior (AP)) and 17 mm AP on the endocanal component. The characteristics of the lesion were compatible with an expansive lesion of indolent growth. Considering the topography and age group, the main diagnostic hypothesis was neuroblastoma.

**Figure 3:**
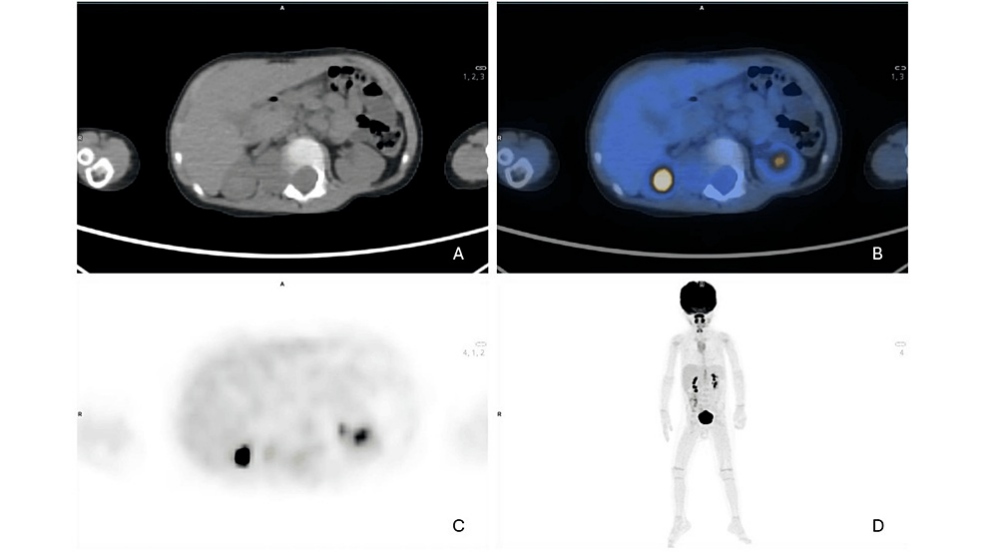
(A and C) Axial view CT. (B) Axial fused PET/CT. (D) Maximum intensity projection, PET/CT imaging of dorsolumbar transition. Study compatible with a high metabolic grade malignant neoplastic lesion centered in the right dorsolumbar paravertebral region with extension and invasion of the spinal canal, requiring histopathological characterization. Small ill-defined perilesional lymph nodes suspected of secondary locations were admitted.

For diagnostic clarification, a positron emission tomography/computed tomography (PET/CT) scan was performed (Figure 3).

**Figure 4:**
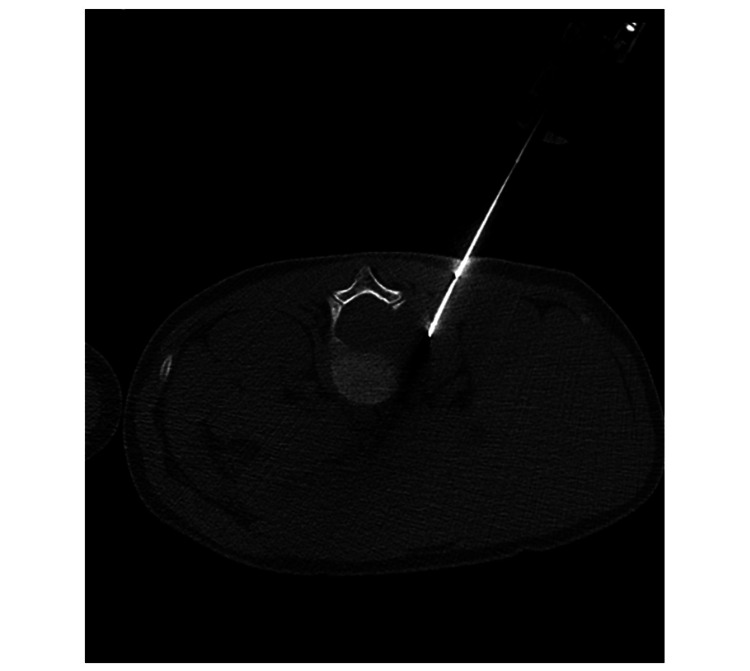
Axial view of the CT-guided biopsy procedure. The histopathological characterization confirmed the diagnosis of paravertebral ganglioneuroma.

For histopathological characterization, a CT-guided biopsy confirmed the diagnosis of paravertebral GN (Figure 4).

Based on these findings, with the parent’s approval, surgery was proposed for tumor resection. In December 2021, surgery was carried out placing the electrodes to monitor somatosensory evoked potentials (SSEP), which at the beginning of the surgery registered a complete absence of motor potentials and only attenuated recording of sensory potentials. The child was in the prone position with slight flexion of the dorsolumbar spine. A median linear incision from D12 to L2 was performed, opening the lumbar fascia and dissection of the cerebral muscular planes of D12, L1, and L2, exposing the right paravertebral extension retroperitoneal. A D12 to L2 laminotomy was performed. The intracanal and extradural tumor mass that was under tension was identified. Through the enlarged foramen of L1-L2, the tumor mass was exteriorized, extending into the vertebral and retroperitoneal space toward the right renal level and in-depth to the level of the vertebral body of D11 and adjacent to the cava vein. Resection of the intracanal tumor began piece by piece with an ultrasonic aspirator, internal decompression of the tumor, and subsequently removing its capsule, partially adhering to the dura mater, but detaching entirely. The tumor had a white appearance, a hard consistency, little vascularization, and a good plane of dissection. Surgery on the paravertebral tumor component was carried out since, like the intracanal component, it presented a good plane and cleavage and allowed its complete resection. Then, the replacement and fixation of the laminotomy piece were performed. At the end of the surgery, monitoring of the SSEP registered an improvement in motor potentials bilaterally and a slight improvement in sensory potentials bilaterally.

Regarding the excised lesion, macroscopically, several nodular formations with a whitish external surface, consisting of yellowish-white tissue, measuring approximately 4 cm in diameter were noted. Microscopically, a neoplasm surrounded in some areas by a fibrous capsule was observed. Elongated cells formed parallel or crisscrossed bundles with eosinophilic cytoplasms with poorly defined boundaries and elongated or wavy nuclei and fine chromatin without significant nuclear atypia or mitotic figures. Ganglion cells were also observed dispersed or forming clusters with abundant eosinophilic or clear and vacuolated cytoplasms and a fine chromatin nucleus with a prominent nucleus, some bi- or multinucleated. The tumor stroma was of collagen and reticulin fibers and there was a focal mononuclear inflammatory infiltrate. The immunohistochemical study showed intense and diffuse positivity of elongated cells for S100 protein and expression of ganglion cells for synaptophysin and neurofilaments. This examination confirmed a ganglioneuroma.

Given that this is a pathology that could produce hormones, it was measured, showing normal levels of catecholamine and vasoactive intestinal peptides.

At the two-month postoperative reassessment, the child was undergoing physiotherapy sessions three times a week, presenting with independent walking with swaying due to grade IV paraparesis, according to the Medical Research Council scale, with a more pronounced deficit in the left lower limb (with a left hanging foot gait), and a widened gait caused also by some atrophy of the crural muscles. The child at this stage was three years old, with sphincter control.

**Figure 5:**
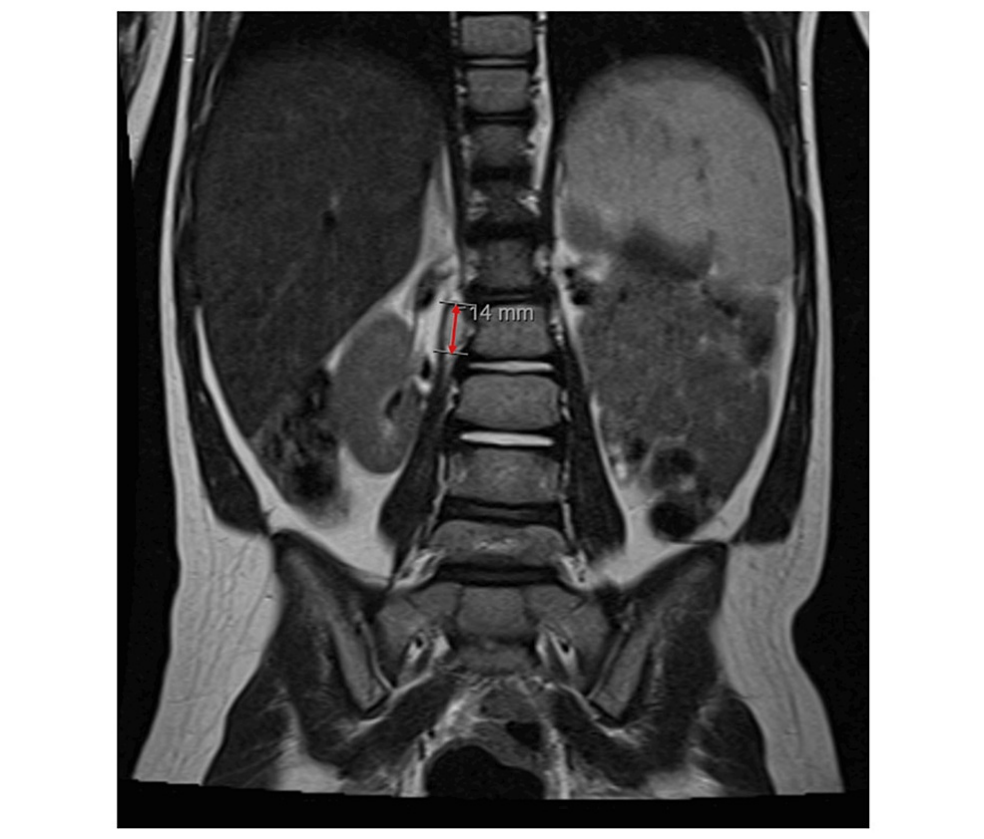
Coronal view of the dorsolumbar follow-up MRI six months after surgery. Almost total removal of the right paravertebral component of the lesion, with only a small right lateral paravertebral formation at the level of the L2 body, suggesting a small residual tumor, at the anterior and internal end of the previously observed tumor. It measures about 12 x 6 x 18 mm (vertical x transversal x anteroposterior). Resolution of the mass effect on the spinal cord.

Six months after surgery, the mother reported a significant improvement in motor development, noting that the child was now running and jumping, while still undergoing motor rehabilitation sessions. On physical examination, nearly symmetric gait, a slight widening of the base of support, and no imbalances were observed. The six-month follow-up MRI can be analyzed in Figure 5.

One year after surgery, the child no longer required frequent physiotherapy and was performing well in school activities. On objective examination, there were no gait alterations, no motor deficits, and the child could perform squats. Due to favorable clinical evolution, annual surveillance of the residual tumor dimensions with MRI was decided by oncology, since it is a tumor with a poor response to chemotherapy and radiotherapy.

Currently, the child is five years old and remains under regular clinical evaluation and annual imaging check-ups with MRI in oncology appointments, as under child health surveillance appointments in primary care.

## Discussion

GN is a well-differentiated benign peripheral tumor that develops from ganglion cells of the posterior mediastinum, retroperitoneum, cervical spine, and adrenal glands [12]. Neurogenic tumors can be classified according to their origin from nerve cells or nerve sheaths. The first group includes GN, gangliogliomas, ganglioneuroblastomas, and neuroblastomas. The second group includes neurilemmomas, neurofibromas, and malignant schwannomas [13]. Eden [12], in 1941, divided tumors like a dumbbell into four categories according to spinal cord and vertebrae involvement: intra- and extradural (type 1); intra- and extradural and paravertebral (type 2); extradural and paravertebral (type 3); foraminal and paravertebral (type 4). In this report, the tumor was extradural and paravertebral on the right, and when passing through the intervertebral foramen, it formed a mass similar to a dumbbell (Figure 2), corresponding to a type 3 tumor, according to Eden's classification. The most common dumbbell-shaped tumor is Schwann cell tumor, while GN is relatively rare [14].

These tumors can secrete hormones such as catecholamines or vasoactive intestinal peptides. In the present case, hormone levels were within normal limits.

Approximately 60% of cases occur in individuals under 20 years of age at the time of diagnosis, with a male-to-female ratio of approximately 2:3. The average age of diagnosis is seven years, contrasting with neuroblastoma, which occurs in children under three years (initial hypothesis after the first MRI in this clinical case, not confirmed subsequently) [4-6].

Clinically, most cases are asymptomatic, leading to frequent incidental diagnoses. Symptoms such as pain, edemas, gastrointestinal problems, neurological issues, or hypertension are not only associated with the tumor size but also with the location of the GN. Edema is common in cervical GN, hypertension in adrenal GN (related to catecholamine hormone production), and neurological symptoms in thoracic or cervical GN [1]. In this case, the neurological symptoms were due to mass compression effects on the medullary cone.

Currently, imaging differentiation between GN and other tumors is challenging. In most cases, surgery is performed with complete tumor resection for histopathological confirmation. Even after complete resection, tumor progression is rare. GNs have a low recurrence rate and good prognosis and do not require chemotherapy, radiotherapy, or other adjuvant treatments. The postoperative complication rate is around 10%, with significant differences depending on the tumor location. Patients should be regularly monitored with ultrasounds and CT scans to detect partial recurrences early after surgery. In this report, a residual right paravertebral mass was observed, requiring regular monitoring of its dimensions [15,16].

The differential diagnosis depends on the location. For paravertebral lesions, the diagnosis includes neuroblastoma and vertebral ganglioneuroblastoma, which can be differentiated by the presence of metastases or calcifications on imaging (more suggestive of these pathologies). Additionally, diagnosis of vertebral schwannoma or neurofibroma should be considered, more commonly seen in middle-aged adults, with lesions centered on the neuronal foramen [5].

It is worth emphasizing the importance of implementing and adhering to the National Program for Child and Youth Health. This program guides child and youth health surveillance appointments, including scheduling key age-specific check-ups and vaccinations, evaluating growth and development with records in the child and youth health booklet, anticipatory care for health promotion and disease prevention, emotional and behavioral disorder prevention, support and stimulation of parental responsibilities, and early detection, monitoring, and referral of alarming situations. During the two-year appointment, warning signs should be evaluated, including throwing objects away, not building anything, not understanding what is said, not uttering intelligible words, showing no interest in surroundings, not establishing contact, not seeking to imitate behaviors, having strabismus, and not walking alone [11]. In this case, a child who attended all recommended key age appointments was able to have early identification of gait changes and received appropriate treatment. It not only reinforces the importance of this care but also serves to remind and raise awareness, particularly among professionals conducting child and youth health surveillance appointments, that such rare situations can occur.

## Conclusions

This case report highlights the importance of adhering to the National Program for Child and Youth Health. It allows close and regular contact between the family and the family doctor, enabling not only the assessment of the child’s growth and development but also the exploration of ideas, expectations, and concerns of the respective parties in all phases.

In this regard, at the two-year-old child’s health assessment, the parents' concerns were identified and a proper physical examination led to the diagnosis and treatment of a rare benign condition with a significant impact on the child’s overall development.
